# Transcriptomic analysis shows that surgical treatment is likely to influence the endometrial receptivity of patients with stage III/IV endometriosis

**DOI:** 10.3389/fendo.2022.932339

**Published:** 2022-08-29

**Authors:** Rui Xiang, Peigen Chen, Zhi Zeng, Huijun Liu, Juan Zhou, Chuanchuan Zhou, Jintao Peng, Haitao Zeng

**Affiliations:** ^1^Reproductive Medicine Center, The Sixth Affiliated Hospital, Sun Yat-sen University, Guangzhou, China; ^2^Department of Gynecology and Obstetrics, The Sixth Affiliated Hospital, Sun Yat-sen University, Guangzhou, China

**Keywords:** endometriosis (EM), surgical treatment, endometrial receptivity, RNA sequencing (RNA-seq), secretory endometrium (SE), immune cell profile

## Abstract

**Background:**

Endometriosis negatively affects fertility, and it is a common disease in assisted reproductive practice. Surgical removal of endometriotic lesions is widely carried out to relieve symptoms and promote fertility. But it is not intensively investigated what changes in the secretory eutopic endometrium of patients with endometriosis after surgery.

**Methods:**

Eighteen patients with stage III/IV endometriosis were included in the study, and they were divided into the untreated group and the treated group (6 vs. 12). Basic clinical data were compared, and transcriptomic data of the secretory eutopic endometrium were analyzed with DESeq2, Cytoscape, ClueGO, CluePedia, and Gene Set Enrichment Analysis (GSEA). CIBERSORT was used to calculate the relative abundance of 22 immune cells in the samples.

**Results:**

We determined 346 differentially expressed genes (DEGs) using DESeq2. These DEGs were used to enrich seven Gene Ontology terms including three associated with immune processes and one correlated to prostaglandin using ClueGO and CluePedia. GSEA enriched 28 Gene Ontology terms in the treated group mainly associated with immune and blood pressure regulation process. Compared to the untreated group, the relative abundance of resting CD4+ memory T cells [0.218 (0.069, 0.334) vs. 0.332 (0.181, 0.429), *P* = 0.022] and the even less abundant memory B cells [0.001 (0.000, 0.083) vs. 0.033 (0.007, 0.057), *P* = 0.049] are significantly decreased in the treated group.

**Conclusion:**

Surgical treatment of stage III/IV endometriosis influences some genes and biological processes related to endometrial receptivity, but more evidence is needed.

## Introduction

Endometriosis is recognized as a chronic estrogen-dependent complex syndrome and inflammatory disease that affects around 5%~10% of women, characterized by the abnormally existing endometrium-like tissues outside the uterus, commonly located on pelvic organs and tissues (1). Endometriosis significantly disturbs female fertility partly due to abnormal endometrial receptivity since studies in human and animal models show defective implantation (2).

Endometrial receptivity, coined as the ability of the endometrium to allow normal implantation, is fundamental for a successful pregnancy and is affected in patients with endometriosis. Multi-omics were applied to identify potential biomarkers for assessing endometrial receptivity. Clinical treatment of endometriosis improves pregnancy rates, and basic research showed abnormal endometrial milieu. For instance, prokineticin-1 (*PROK1*), which is important in the vascular function of peri-implantation endometrium and early pregnancy, is significantly less expressed in the eutopic endometrium of patients with endometriosis (3). Other factors that are associated with decidualization and implantation such as L-selectin ligand, *ανβ3* integrin, and leukemia inhibitory factor (*LIF*) are also decreased in the eutopic endometrium (4–6).

Surgery that removes ectopic endometrial tissue is a common measure to relieve symptoms and promote fertility. Some evidence shows that fertility is improved after surgery (7, 8), but the changes in the eutopic endometrium after surgery are not well-illustrated. We hereby conducted this research to preliminarily discover the effect of surgery on the secretory eutopic endometrium.

## Materials and methods

### Criteria of patients included in the research groups

Six patients in the untreated group were diagnosed with endometriosis by ultrasound or magnetic resonance imaging (MRI), as endometrioma exists and did not receive surgical treatment for endometriosis. Twelve patients in the treated group were confirmed to have endometriosis during laparoscopic or transabdominal surgery in less than 24 months before endometrial sampling, and the endometriotic lesions were removed during surgery. All 18 patients were of stage III/IV according to the revised American Fertility Society (r-AFS) classification. The exclusion criteria are as follows: 1) follicle stimulation hormone (FSH) >12 mIU/ml and anti-Müllerian hormone (AMH) <1.1 ng/ml; 2) abnormal uterine cavity morphology by ultrasound, MRI, or hysteroscopy; 3) signs of malignancies.

### Endometrial preparation and sample collection protocol

Patients received 2 mg of estradiol valerate (Progynova, Bayer Pharma AG, Berlin, Germany) twice daily for 28 days starting on the second day of menstrual bleeding. From the 15th day onward, 10 mg of dydrogesterone (Duphaston, Abbott Biologicals B.V., Netherlands) twice daily was applied.

Endometrial sampling was conducted on P+5 (the 5th day of Duphaston administration) using a sterile endometrium sampler kit with a separate package (Type I, Run Ting). The patient was kept in the lithotomy position and received vulvar and vaginal disinfection with the introduction of the disinfected speculum, and a sterile swab was inserted into the cervical canal to avoid contamination. Then, a disposable sterile endometrial sampler (Yikon Inc.) was introduced into the uterine cavity to suck the endometrium. After that, the external surface of the catheter was cleaned with a sterile gauze and half of its content was transferred to cryotubes containing 1.5 ml of tissue preservation solution (XK-039-3, Yikon Inc.) at -20°C for future RNA extraction and PCR test, and the other half was stored in formalin for hematoxylin and eosin staining and immunohistochemistry staining for CD138. Chronic endometritis (CE) is determined as at least one plasma cell is found in the endometrial biopsy using immunohistochemistry staining for CD138 (9, 10).

### RNA extraction and RNA sequencing procession

Total RNA was extracted using RNeasy Micro Kit (74004, Qiagen) according to the manufacturer’s instructions. Then, RNA quantitative detection was conducted with Qubit RNA HS Kit (Q32855; Thermo Fisher Scientific). Agilent Bioanalyzer 2100 (Agilent) was used to check the integrity of the extracted total RNA. Only samples whose RNA integrity number (RIN) is greater than 7 were considered qualified samples and used for subsequent testing.

Next, RNA reverse transcription and amplification were conducted using MALBAC^®^ Platinum Single Cell RNA Amplification Kit (KT110700796; Xukang Co., Ltd.). The positive and negative controls were 500 ng of high-quality host total RNA and ultrapure water, respectively. After that, 1 µl cDNA was 10-fold diluted and detected with Agilent Bioanalyzer 2100 (Agilent).

Finally, the library was constructed using gene sequencing and library preparation kit (XK-038, Xukang Co., Ltd.) according to the manufacturer’s instructions. Following purification, the library was quantified with Qubit dsDNA HS kit (Q32584, Invitrogen). According to the quantitative results of Qubit, each sample was taken from 10 ng library and mixed in equal proportions, followed by Qubit quantitative detection again. Pair-end sequencing of the products was performed on the NextSeq CN500 platform (Illumina).

### RNA extraction, quantitative real-time PCR, and public database search

Total RNA was extracted from five samples from the untreated group and 12 samples from the treated group using RNeasy Micro Kit (Qiagen, MD, USA; Cat# 74004) according to manufacturer’s instructions. Reverse transcription of first-strand complementary DNAs (cDNAs) was carried out using HiScript III RT SuperMix for qPCR (Vazyme, Nanjing, China, Cat# R323-01). Quantitative PCR (qPCR) was conducted using 2× RealStar Green Power Mixture (Genstar; Cat# A311-101) on a Roche LightCycler 480 II (Roche Diagnostics, Mannheim, Germany). The expression of glyceraldehyde-3-phosphate dehydrogenase (GAPDH) was chosen as an endogenous control. The primers used were as follows:

*HOXB2*: forward: GATGAAAGAGAAGAAATCCGCC,

reverse: AAGTGGAATTCCTTCTCCAGTT;

*GAPDH*: forward: GGTCGGAGTCAACGGATTT,

reverse: CCAGCATCGCCCCACTTG.

The Human Protein Atlas (11) is used for the *HOXB2* gene expression feature identification.

### RNA sequencing analysis procession

Firstly, FastQC v.0.11.9 (http://www.bioinformatics.babraham.ac.uk/projects/fastqc/) was used to evaluate fastq files, and then the files were processed with trim_galore (http://www.bioinformatics.babraham.ac.uk/projects/trim_galore/) to remove reads containing adapters, more than 10% unknown nucleotides (N), and more than 50% of low-quality (Q-value ≤20) bases. Next, HISAT2 (12) was used to map the processed paired-end reads to the human genome GRCh38 from Gencode v26. FeatureCount (13) was used to generate the count value of gene expression in the form of a matrix. After that, principal component analysis was done with TPM (Transcripts Per Kilobase of exon model per Million mapped reads) value normalized from the matrix. The differentially expressed genes (DEGs) between the two groups were selected by using DESeq2 R package (14) with the *P* value <0.05 and log fold change (logFC) >1 as the cutoff. Then, functional enrichment analysis of DEGs was performed with ClueGO v.2.5.8 (15) and CluePedia 1.5.8 (16) plugins of Cytoscape v.3.9.1 (17). GSEA 4.2.1 (http://www.gsea-msigdb.org/gsea/index.jsp) was used to conduct functional enrichment analysis of whole gene expression features.

### Calculation of endometrial immune cell abundance

CIBERSORT (18) was used to estimate the abundance of 22 types of immune cells in the endometrium.

### Statistical analysis

Clinical characteristics of participants, including age, body mass index (BMI), FSH, and AMH, are presented as the mean ± standard deviation (SD) and were compared between groups using the *t*-test for equality of means. The relative abundance of immune cells is presented as the median (range) and was compared between groups using the nonparametric Mann–Whitney U-test. CE rate is presented as n% (positive/all) and was compared between groups using the Fisher’s exact test. Above statistical analysis is conducted with SPSS 22.0 software (IBM Corporation, Armonk, NY, USA). The relative mRNA expression ratio was compared between groups using the nonparametric Mann–Whitney U-test by GraphPad Prism 9.0.0 software (Bethesda, MD, USA). Statistical significance was set at *P* value <0.05.

## Results

### Clinical features of enrolled patients

The age, BMI, FSH, AMH, and CE rates are demonstrated in **Table 1**.

**Table 1:** Basic information of the untreated and treated groups.

Items	The untreated	The treated	*P* value
No. of patients	6	12	–
Age (years)	34.17 ± 3.76	33.08 ± 2.23	0.451
BMI	21.07 ± 1.40	20.99 ± 2.43	0.945
FSH (IU/L)	6.17 ± 2.77	6.62 ± 2.06	0.698
AMH (ng/ml)	4.06 ± 1.70	2.58 ± 1.23	0.050
CE	16.67% (1/6)	8.33% (1/12)	>0.999

BMI, body mass index; FSH, follicle stimulating hormone; AMH, anti-Müllerian hormone; CE, chronic endometritis.

### Transcriptomic features of the secretory eutopic endometrium in between the untreated and treated groups

**Figure 1:**
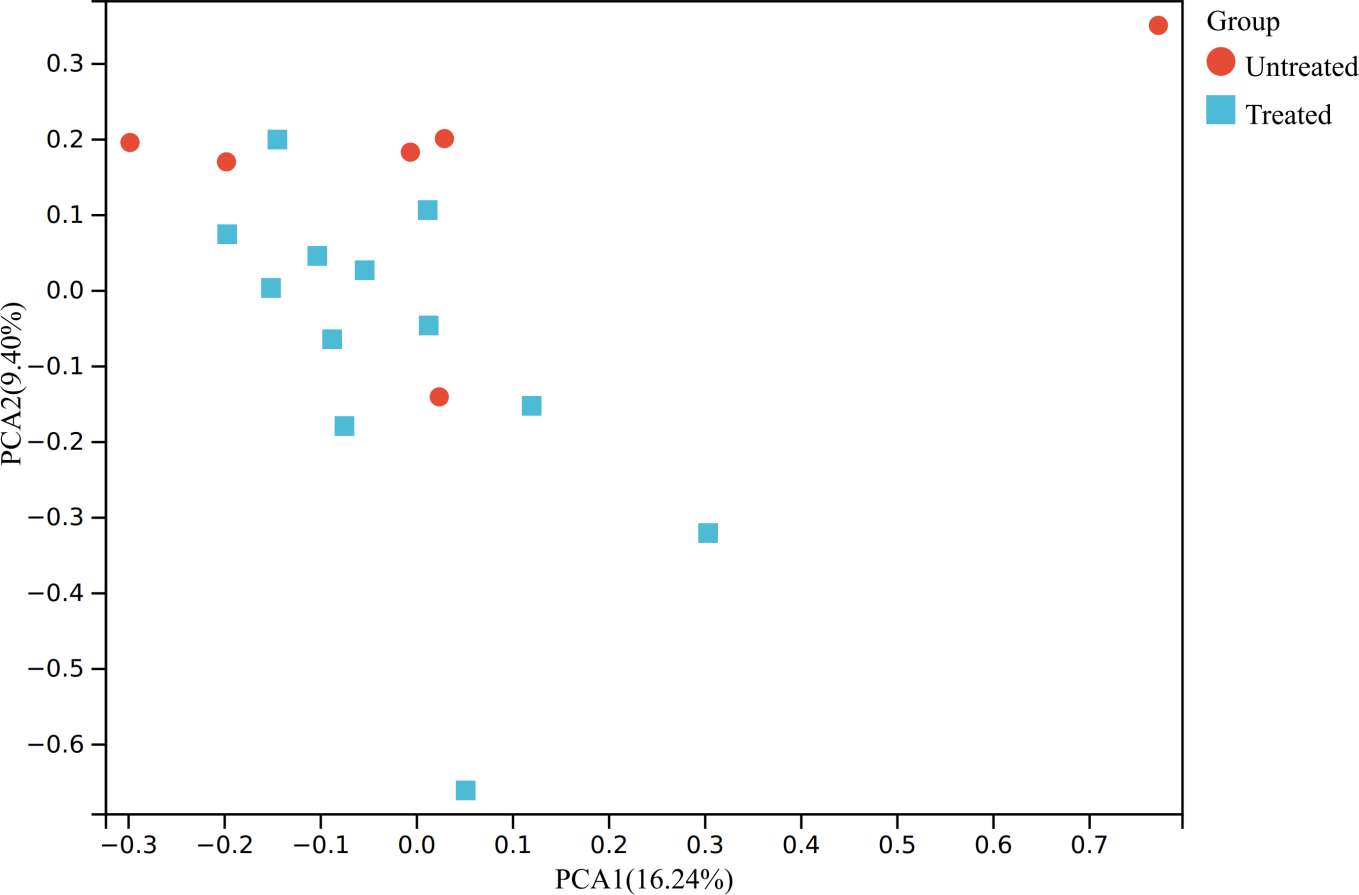
PCA of the transcriptomic data. The raw expression matrix was normalized to TPM before PCA. PCA, principal component analysis; TPM, Transcripts Per Kilobase of exon model per Million mapped reads; Untreated, the untreated group; Treated, the treated group.

We first conducted PCA that shows relatively obvious discrimination between the two groups (**Figure 1**).

**Figure 2:**
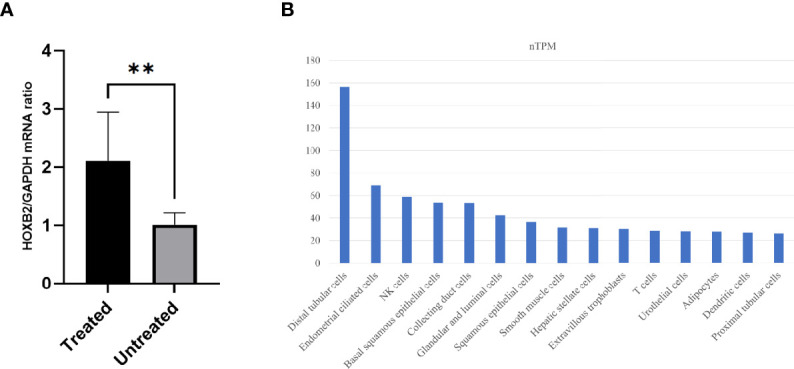
The expression of HOXB2. **(A)** Relative mRNA expression of HOXB2 in the treated (T) and untreated (UT) groups by quantitative real-time PCR. **P value = 0.006. **(B)** Top 15 cell types with high expression levels of HOXB2. Raw data are from the human protein atlas (www.proteinatlas.org). Untreated, the untreated group; Treated, the treated group.

Then, we used DESeq2 to determine the DEGs between the groups. A total of 346 DEGs were filtered with logFC >|1| and *P* value <0.05. Among these DEGs, 140 and 206 genes are upregulated and downregulated in the treated group, respectively. The top 10 upregulated and downregulated genes with high expression levels are exhibited in **Table 2**. All 346 DEGs are presented in **Supplementary Material 1**. Of the 20 top expressed DEGs, we found *HOXB2* to be of importance, since it is a downstream gene of type I interferon (IFN) response and we validated its expression by qPCR (**Figure 2A**). We then searched for the expression feature of *HOXB2* in the human protein atlas database, and it turns out that *HOXB2* expresses highly in endometrial ciliated cells, natural killer (NK) cells, and T cells (**Figure 2B**).

**Table 2:** Top 10 upregulated and downregulated genes with a high expression level.

ENSEMBL ID	Gene Symbol	Gene Name	Biotype	Base Mean	logFC	*P* value
Up-regulated genes						
ENSG00000157613	CREB3L1	cAMP-responsive element-binding protein 3 like 1	Protein coding	915.372	1.109	0.004
ENSG00000100075	SLC25A1	Solute carrier family 25 member 1	Protein coding	259.557	1.165	0.001
ENSG00000179271	GADD45GIP1	GADD45G interacting protein 1	Protein coding	120.709	1.119	<0.001
ENSG00000078808	SDF4	Stromal cell derived factor 4	Protein coding	108.906	1.289	<0.001
ENSG00000102760	RGCC	Regulator of cell cycle	Protein coding	65.688	1.583	0.020
ENSG00000173917	HOXB2	Homeobox B2	Protein coding	63.869	1.042	0.006
ENSG00000157933	SKI	SKI proto-oncogene	Protein coding	56.638	1.085	<0.001
ENSG00000170624	SGCD	Sarcoglycan delta	Protein coding	50.021	1.057	0.029
ENSG00000051523	CYBA	Cytochrome b-245 alpha chain	Protein coding	43.135	1.053	0.001
ENSG00000176463	SLCO3A1	Solute carrier organic anion transporter family member 3A1	Protein coding	42.782	1.325	<0.001
Down-regulated genes						
ENSG00000134333	LDHA	Lactate dehydrogenase A	Protein coding	4,270.837	-1.017	<0.001
ENSG00000165507	DEPP1	DEPP1 autophagy regulator	Protein coding	2,169.889	-1.850	0.002
ENSG00000100342	APOL1	Apolipoprotein L1	Protein coding	2,075.956	-1.111	<0.001
ENSG00000189143	CLDN4	Claudin 4	Protein coding	1,989.718	-1.147	<0.001
ENSG00000159167	STC1	Stanniocalcin 1	Protein coding	1,761.674	-1.441	0.028
ENSG00000150347	ARID5B	AT-rich interaction domain 5B	Protein coding	1,452.366	-1.049	0.008
ENSG00000067082	KLF6	Kruppel-like factor 6	Protein coding	1,253.342	-1.033	0.001
ENSG00000122884	P4HA1	Prolyl 4-hydroxylase subunit alpha 1	Protein coding	1,146.017	-1.028	<0.001
ENSG00000196352	CD55	CD55 molecule	Protein coding	773.112	-1.035	0.004
ENSG00000162896	PIGR	Polymeric immunoglobulin receptor	Protein coding	681.500	-1.157	0.006

### Functional analysis of the transcriptomic features

**Figure 3:**
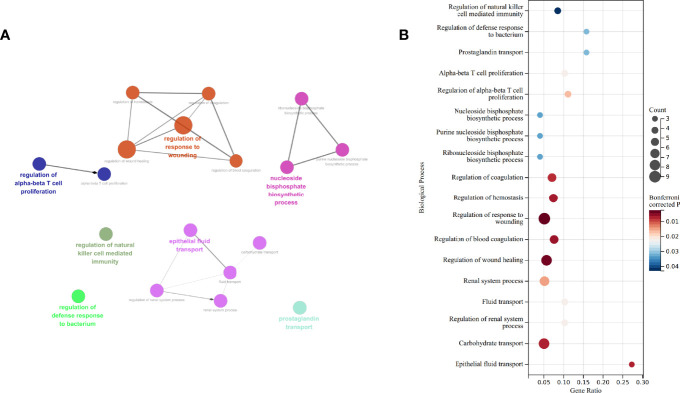
GO enrichment by ClueGO and CluePedia. **(A)** Network of biological process enriched. **(B)** Gene counts, gene ratio, and *P* value after Bonferroni correction of each GO: biological process terms. GO, gene ontology.

Functional analysis derived from ClueGO suggests that “regulation of αβT cell proliferation,” “regulation of natural killer cell mediated immunity,” “regulation of defense response to bacterium,” “regulation of response to wounding,” “epithelial fluid transport,” “nucleoside biphosphate biosynthetic process,” and “prostaglandin transport” are different between the untreated and treated groups (**Figure 3A**). Of the seven Gene Ontology (GO) terms, three are associated with immune processes. Gene counts, gene ratio, and Bonferroni-corrected *P* value of each term are shown in **Figure 3B**.

To have a broader view of the potential changes based on the whole transcriptomic features, we ran GSEA and enriched 28 GO terms in the treated group, including 17 terms of biological process, 8 terms of molecular function, and 3 terms of cellular component (**Table 3**). Of these GO terms, “defense response to Gram negative bacterium,” “antibacterial humoral response,” “humoral immune response mediated by circulating immunoglobulin”, “immunological memory process”, “immunoglobulin receptor binding”, “antigen binding”, “peptide antigen binding”, “immunoglobulin complex”, “T cell receptor complex,” and “MHC protein complex” correlate with immune process.

**Table 3:** Enriched GO terms of 12 samples in the treated group by GSEA.

GO terms	NES	*P* value	FDR
Biological Process			
Regulation of systemic arterial blood pressure by renin angiotensin	-2.036	<0.001	0.080
Serotonin receptor signaling pathway	-2.003	<0.001	0.087
Hepatocyte differentiation	-1.936	<0.001	0.105
Regulation of systemic arterial blood pressure by hormone	-1.934	0.008	0.095
Regulation of systemic arterial blood pressure mediated by a chemical signal	-1.862	0.010	0.130
Regulation of systemic arterial blood pressure by circulatory renin angiotensin	-1.849	0.026	0.120
Defense response to Gram-negative bacterium	-1.786	<0.001	0.136
Sensory perception of smell	-1.777	<0.001	0.136
Positive regulation of organic acid transport	-1.736	0.006	0.154
Detection of chemical stimulus	-1.710	<0.001	0.174
Vasodilation	-1.670	0.015	0.214
Antibacterial humoral response	-1.670	0.011	0.206
Phagocytosis recognition	-1.659	0.015	0.203
Humoral immune response mediated by circulating immunoglobulin	-1.648	<0.001	0.209
Regulation of systemic arterial blood pressure	-1.646	<0.001	0.204
Immunological memory process	-1.641	0.015	0.203
Icosanoid secretion	-1.640	0.008	0.198
Molecular Function			
Serotonin receptor activity	-2.191	<0.001	0.030
Olfactory receptor activity	-1.978	<0.001	0.090
NAD^+^ nucleosidase activity	-1.898	<0.001	0.111
Immunoglobulin receptor binding	-1.853	0.016	0.126
Carbohydrate derivative transmembrane transporter activity	-1.844	<0.001	0.116
Antigen binding	-1.804	<0.001	0.137
Peptide antigen binding	-1.769	0.023	0.136
G protein-coupled amine receptor activity	-1.666	0.013	0.203
Cellular Component			
Immunoglobulin complex	-2.313	<0.001	0.014
T-cell receptor complex	-1.804	<0.001	0.129
MHC protein complex	-1.745	0.014	0.153

NES, normalized enrichment score; FDR, false discovery rate.

Several terms associated with blood pressure regulation such as “regulation of systemic arterial blood pressure by renin angiotensin”, “serotonin receptor signaling pathway”, “regulation of systemic arterial blood pressure by hormone”, “regulation of systemic arterial blood pressure mediated by a chemical signal”, “regulation of systemic arterial blood pressure by circulatory renin angiotensin”, “vasodilation”, “regulation of systemic arterial blood pressure”, and “serotonin receptor activity” are also enriched.

### Calculated immune cell abundance in the secretory eutopic endometrium in between the untreated and treated groups

**Figure 4:**
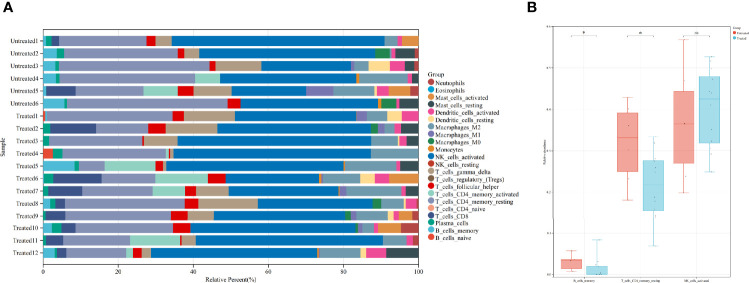
Immune cell abundance and comparison of certain immune cells between the groups. **(A)** CIBERSORT-calculated relative abundance of 22 different immune cells in the 18 samples. **(B)** Comparison of memory B cells, resting CD4+ memory T cells, and activated NK cells in the treated and untreated groups. *P value <0.05. ns, not significant. Untreated, the untreated group; Treated, the treated group.

To further investigate the immunological changes in the endometrium, we used CIBERSORT to estimate the relative percent of 22 types of immune cells. Activated NK cells and resting CD4+ memory T cells comprise the majority of the immune cell population in the secretory eutopic endometrium (**Figure 4A**).

Then, the immune cell populations were compared between the groups, three of them are shown in **Figure 4B**. Activated NK cells are not significantly different [0.424 (0.248, 0.526) in the treated group vs. 0.365 (0.198, 0.568) in the untreated group, *P* = 0.574]. While the less abundant resting CD4+ memory T cells and the even less abundant memory B cells are significantly decreased in the treated group [0.218 (0.069, 0.334) vs. 0.332 (0.181, 0.429), *P* = 0.022, and 0.001 (0.000, 0.083) vs. 0.033 (0.007, 0.057), *P* = 0.049, respectively]. The comparison of all of the immune cells is concluded in **Supplementary Material 2**.

## Discussion

In this study, we applied RNA-seq analysis and CIBERSORT to profile the endometrial environment during the secretory phase after surgical treatment of endometriosis. We found several DEGs and biological processes that could be associated with receptivity. Also, altered function and abundance of certain immune cells are speculated to be related to the possibly differed endometrial microbiota after surgery.

### Changes identified at the gene expression level

The expression of certain genes associated with endometriosis and receptivity is altered after surgery.

*HOXB2*, a relatively less studied member of the homeobox genes in the endometrium is upregulated in type I IFN response (19), and one study pointed out that the response to IFN signaling is dysregulated in the eutopic endometrium of patients with adenomyosis during the secretory phase (20). IFNs bind to specific membrane receptors to exert biological activities. Upregulated transcription of IFN-α in the human endometrium during the window of implantation (21) and the effect of type I IFN-τ on receptivity and implantation in the endometrium of the ruminant animal (22) indicate the importance of IFNs in fertility. IFNs also play an important role in immune modulation, including the adaptive immune response (23). As a downstream target gene of type I IFN, the upregulated *HOXB2* expression is a putative implication of the altered IFN signaling in the endometrium of patients with endometriosis after surgery. *CREB3L1* that is identified as a target gene of the progesterone receptor is decreased in the eutopic endometrium of patients with endometriosis, and it regulates the phosphorylation of extracellular signal-regulated kinase (ERK)1/2 in the decidualization process (24). The transcription level of *CLDN4* is significantly higher in the endometrium of idiopathic infertility and minimal endometriosis, indicating that *CLDN4* overexpression might be negatively linked to endometrial receptivity (25). *STC1* serves in many processes (26), and it correlates with receptivity markers during the window of implantation (27). In patients with endometriosis, *STC1* is upregulated in the mid-secretary eutopic endometrium (28).

In our results, *CREB3L1* and *HOXB2* are upregulated after surgery, while *CLDN4* and *STC1* are downregulated. These alterations might be important in remodeling receptivity.

### Changes identified at the biological process level

Several biological processes are interconnected and found to be altered after surgery.

Prostaglandins (PGs) are bioactive in tissues and organs. PGE2 is observed to increase during the luteal phase in the animal uterus, and this hints that it might be associated with luteal function and implantation. PGE2 induces LH receptor expression on the corpus luteum through receptor EP2 (29, 30), and it increases the blood supply of the uterus and ovary by increasing a vasodilator called nitric oxide *via* receptor EP4 (31). In a recent review, the authors hypothesized that PGE2 prevents luteolysis by playing a role in the P4 secretion stimulated by estradiol (E2) and IFN-τ, and the latter is a member of type I IFNs (32). In goat endometrial stromal cells, it has been validated that IFN-τ could increase the ratio of PGE2/PGF2α *via* JAB1 and the unfolded protein response (UPR) mainly by regulating the amount of PGE2 rather than PGF2α (33). As the increased expression of *HOXB2* hints, this process is likely to be the underlying mechanism that alters luteal function.

PGE2 is also found to regulate the *CXCR4* expression through the epidermal growth factor receptor (EGFR)-phosphatidyl inositol-3 kinase (PI3K) and ERK1/2 pathway *via* prostaglandin E receptor 2 (PTGER2) in the endometrium (34–37). One study confirmed that *CXCR4* expression is especially increased at the apposition site (38). *CXCR4* is vital in embryonic vasculogenesis (39), which might be crucial in placenta attachment. Furthermore, the PI3K/ERK1/2 pathways enhance the growth, proliferation, differentiation, and survival of endometrial cells and embryos (40). PGE2 could also help increase the expression of *ανβ3* integrin, thus helping embryo adhesion (41). Fetal–maternal crosstalk between the embryo and the endometrium is essential in successful pregnancy, and PGE2 is widely involved in this process. Our analysis suggests that “prostaglandin transport” is altered after surgery, which might contribute to the altered response of the endometrium to the embryo and luteal function, as PGE2 might function in implantation by means that are mentioned above.

### Changes identified at the immune environment level

The endometrium contains different kinds of immune cells and relative molecules, which are important during implantation and pregnancy.

A PGE2–myocyte enhancer factor 2A (MEF2A) axis in type I IFN induction is introduced recently. Research shows that PGE2 interferes with lipopolysaccharide (LPS)-mediated activation of ERK5 that is a known transcriptional partner of MEF2 and thus affects inflammatory gene expression (42). Given the previously mentioned regulation of IFN-τ on PGE2, it can be inferred that the PG and IFN response in the endometrium interact mutually and they maintain balance in an accurate manner, and surgery might influence that regulation process.

Implantation correlates with innate immune cells in the endometrium. Although our analysis did not show significant alteration of several innate immune cells in relative quantity after surgery, “regulation of αβT cell proliferation” and “regulation of natural killer cell mediated immunity” are enriched in the functional enrichment. In patients with endometriosis who obtained successful implantation, uterine NK progenitor cell populations are markedly higher than those in patients who have failed implantation (43). Uterine NK cells secrete angiogenic growth factor, contributing to decidualization and formation of spiral arteries, which makes them important in early pregnancy (44). However, there is evidence that NK cells are not essential for pregnancy (45). The ratio of T helper type 1 (Th1) cytokines over T helper type 2 (Th2) cytokines is related to fetal acceptance. Inflammatory Th1 immunity is dominant at the peri-implantation stage, it could benefit the invading of trophoblasts (46). Interestingly, there is evidence that type I IFN response works in the regulation of NK cells and T cells. In direct and indirect activation of NK cells, type I IFN could activate NK cells through the IFN-activating receptor on NK cells or dendritic cells are first activated by type I IFN, then they activate NK cells by trans-presentation of interleukin (IL)-15 to IL-15 receptors on NK cells, respectively (47). As for T cells, type I IFN could also alter the αβT-cell population by inducing an immune response of dendritic cells *via* both IL-15 and the IL-15R α-chain (48). Exposure to IFN-α/β results in increased expression of genes associated with cell proliferation and cell survival (49), which could be the mechanism of αβT-cell proliferation. Type I IFN leads to Th1 induction and Th2 restriction, which might contribute to better implantation (50). Due to the lack of adequate subjects, the function of T cells and NK cells after surgery needs to be further elucidated.

Implantation might correlate with adaptive immune cells in the endometrium as well. Memory B cells and resting CD4+ memory T cells are less in the treated group. We also find “antibacterial humoral response,” “humoral immune response mediated by circulating immunoglobulin,” and “immunological memory process” are enriched in the treated group. Higher IL-2 seems to be adverse to pregnancy, since it induces increased NK activity of decidual NK cells (51) that leads to a less tolerant endometrial environment, and application of IL-2 causes fetal development inhibition in mice (52). Surgical treatment could reduce serum IL-2 (53), which might facilitate receptivity. CD4+ memory T cells are typical IL-2-producing cells (54), thus their reduction in the relative population might facilitate a healthier milieu for implantation. Both CD4+ memory T cells and memory B cells are generated in the adaptive immune process, so their abundance could be closely related to the microbes in the uterine cavity. Endometrial microbiota seems to be more diverse in patients with endometriosis (55). One study found a complete absence of *Atopobium* in the vaginal and cervical microbiota and an increase of potentially pathogenic *Gardnerella*, *Streptococcus*, *Escherichia*, *Shigella*, and *Ureoplasma* in the cervical microbiota in III/IV endometriosis (56). Another study categorized the endometrial microbial composition as either *Lactobacillus*-dominant (LD, >90% *Lactobacillus* spp.) or non-*Lactobacillus*-dominant (NLD, <90% *Lactobacillus* spp. with >10% of other bacteria), and NLD microbiotas are associated with adverse reproductive outcomes (57). Since the female genital tract is consecutively unobstructed and relatively short, the endometrial microbiota possibly has a crosstalk with that in the lower tract. Clinical administration of a broad-spectrum antibiotic significantly decreased several kinds of microbiomes (58). The endometrial microbiota might alter after surgery, since antibiotics were applied during the surgery and thus caused altered adaptive immune cell proportions. Based on these clues, it could be inferred that adaptive immune cells might be a potential marker of endometrial microbiotic milieu due to their interaction with the microbiota, and the microbiota could interfere with endometrial receptivity indirectly by affecting the adaptive immune cells.

Upon these findings and speculation, we hypothesized that surgical treatment might modify immune components and the endometrial microbiota, leading to the alteration of innate and adaptive immune cells. Further investigation of immune factors and endometrial microbiota after surgery is needed to prove this.

## Data availability statement

The datasets presented in this study can be found in online repositories. The names of the repository/repositories and accession number(s) can be found below: National Genomics Data Center, accession number HRA002724 (https://ngdc.cncb.ac.cn/gsa-human/browse/HRA002724).

## Ethics statement

This study was reviewed and approved by The Ethics Committee of the Sixth Affiliated Hospital of Sun Yat-Sen University. All methods were carried out in accordance with the Declaration of Helsinki and the ethical guidelines and regulations of the Ethics Committee of the Sixth Affiliated Hospital of Sun Yat-Sen University. The patients/participants provided their written informed consent to participate in this study.

## Author contributions

RX, JP, and HZ proposed the idea and designed the research. ZZ, HL, JZ, JP, and HZ collected clinical data and tissue samples of the patients. RX, PC, ZZ, and CZ analyzed and interpreted the transcriptomic data. RX drafted the manuscript. JP and HZ did the revision. All authors read and approved the final manuscript.

## Funding

This work was supported by the National Key Research and Development Program of China (grant no. 2021YFC2700503).

## Acknowledgments

We appreciate Xukang Medical·Yikon Genomics for helping with the sequencing process.

## Conflict of interest

The authors declare that the research was conducted in the absence of any commercial or financial relationships that could be construed as a potential conflict of interest.

## Publisher’s note

All claims expressed in this article are solely those of the authors and do not necessarily represent those of their affiliated organizations, or those of the publisher, the editors and the reviewers. Any product that may be evaluated in this article, or claim that may be made by its manufacturer, is not guaranteed or endorsed by the publisher.
